# The role of B cell antigen receptors in mantle cell lymphoma

**DOI:** 10.1186/s13045-017-0533-9

**Published:** 2017-10-17

**Authors:** Michael Fichtner, Martin Dreyling, Mascha Binder, Martin Trepel

**Affiliations:** 1 Department of Physiology and Medical Physics, Royal College of Surgeons in Ireland, St. Stephen’s Green, Dublin 2, Ireland; 2 Department of Oncology and Hematology, University Medical Center Hamburg-Eppendorf, Martinistr. 52, 20246 Hamburg, Germany; 3 Department of Medicine III, University Hospital LMU Munich, Marchioninistr. 15, 81377 Munich, Germany; 4 Department of Hematology and Oncology, Augsburg Medical Center, Stenglinstr. 2, 86156 Augsburg, Germany

**Keywords:** B cell receptor, Mantle cell lymphoma, Superantigens, Lymphomagenesis, B cell receptor inhibitors

## Abstract

Mantle cell lymphoma (MCL) is characterized by an aggressive clinical course and secondary resistance to currently available therapies in most cases. Therefore, despite recent advances in the treatment of this disease, it is still considered to be incurable in the majority of cases. MCL B cells retain their B cell antigen receptor (BCR) expression during and after neoplastic transformation. BCRs in MCL show distinct patterns of antigen selection and ongoing BCR signaling. However, little is known about the involved antigens and the mechanisms leading to lymphomagenesis and lymphoma progression in MCL. Recent preclinical and clinical studies have established a crucial role of the BCR and the potential of inhibiting its signaling in this disease. This has established the B cell antigen receptor signaling cascade as a very promising therapeutic target to improve outcome in MCL alone or in combination with chemo-immunotherapy in recent years.

## Background

The adaptive human immune system is able to recognize nearly any possible antigen even if it was never encountered before [1, 2]. This high variability is mediated by cell clone-specific, adaptive receptors on B and T cells, called B cell receptors (BCRs) and T cell receptors (TCRs). The development of B and T cells includes the introduction and repair of deoxyribonucleic acid (DNA) double strand breaks to form functional receptors [3]. During this process, erroneous DNA recombination might lead to overexpression of proto-oncogenes, resulting in uncontrolled proliferation of single lymphocytes, eventually transforming into lymphoma [4]. Almost 90% of these neoplasms derive from B cells [5, 6]. Despite the fact that the term Non-Hodgkin lymphoma is still widely used, it has been abandoned in the 2016 revision of the World Health Organization classification of lymphomas. Therefore, we use the currently accepted term of mature B cell neoplasm throughout this review [7].

Mantle cell lymphoma (MCL), accounts for 3–10% of all lymphomas in Europe and the United States [8–10]. The median survival in the overall population of MCL patients is unsatisfying with no plateau in Kaplan Meier survival curves. Similar to most lymphomas, MCLs occur predominantly in the elderly with a median age at diagnosis of 65 years and is more frequent in males (ratio 3–4:1) [10, 11]. MCL has several features clearly differentiating it from other lymphomas. Besides its distinct morphology and immunophenotype, it has a pathognomonic chromosomal translocation, t(11;14) which causes a fusion of the cyclin D1 gene to the immunoglobulin heavy chain promoter leading to constitutive expression of cyclin D1. This is a diagnostic hallmark of the disease and of high pathobiological relevance as cyclin D1 plays a major role in cell cycle control and therefore in proliferation (see below). MCL also has a distinct clinical course and is frequently diagnosed in advanced stages. Except for a few indolent cases, MCL typically has a rapid growth requiring immediate treatment, which places MCL in clinical proximity to other aggressive lymphomas such as diffuse large B cell lymphoma (DLBCL). It also responds to similar immune-chemotherapeutic treatments (e.g., a combination of the anti-CD20 antibody rituximab and cyclophosphamide, doxorubicin, vincristine, and prednisone (R-CHOP)). Such treatment paradigms in MCL have been refined in recent years, and the clinical outcome has been significantly improved [12]. In fact, younger and fit patients treated upfront with intensified protocols like R-CHOP/R-DHAP (rituximab, dexamethasone, high-dose AraC, cisplatin) followed by high-dose chemotherapy with subsequent autologous stem cell transplantation or R-Hyper-CVAD/MA (rituximab with cyclophosphamide, doxorubicin, vincristine, dexamethasone, methotrexate, AraC) have a median progression-free survival of more than 7 years [13–16]. Very recent data suggest that survival after autologous stem cell transplantation can be further improved by rituximab maintenance therapy over 3 years [17]. Also, even elderly patients achieve ongoing remissions due to better tolerated R-bendamustine [18]. Nevertheless, in contrast to other aggressive lymphomas, after achieving remission of the disease, MCL usually relapse within several years. In this situation, treatment options are limited. Previously, only few patients could be salvaged with very aggressive treatments including allogeneic stem cell transplantation [19]. In recent years, however, several molecularly targeted therapeutic strategies have been introduced that have further improved the outcome of relapsed MCL patients not eligible for or prior to allogeneic stem cell transplantation (see below). In this regard, targeting the B cell receptor signaling pathway in MCL has been the most promising step forward, both in view of understanding the pathobiology of this disease as well as in view of advancing its treatment. These two issues will be reviewed in the following sections.

## Overview on BCR development

The B cell receptor consists of a membrane-bound immunoglobulin that is associated with the transmembrane proteins CD79a and CD79b [20]. The latter facilitate signal transduction into the cell via phosphorylation of their cytoplasmic immunoreceptor tyrosine-based activation motifs (ITAMs) after binding of the ligand to the immunoglobulin [20–22]. The immunoglobulin itself consists of two identical light and two identical heavy chains which together form a Y-shaped molecule that harbors two identical antigen-binding sites at the N-terminal ends. Antigen binding is facilitated by three highly variable regions, called complementarity determining regions (CDRs), which are located in the variable domains of each immunoglobulin chain [23]. In contrast to most other proteins, the gene sequence of the variable immunoglobulin regions is not directly encoded in the germline. Instead, the development of a functional BCR requires multiple chromosomal rearrangements and targeted induction of point mutations to generate a very specific BCR with high affinity against a foreign antigen but no reactivity against self-antigens [3, 23]. This process includes a random rearrangement of specific heavy chain gene segments called V(ariable)-, D(iversification), and J(oining)-gene segment as well as V- and J-gene segments of the light chain [24]. The CDR3-regions of the heavy and light chains are formed independently of antigen contact by the combination of the V-(, D-) and J-gene segments in the bone marrow (reviewed in [23]). After successful recombination of the gene segments on one allele, the other allele becomes silenced (allelic exclusion) to ensure that every B cell is committed to only one distinct BCR [25, 26].

With a recombined BCR, the naïve B cells migrate towards the secondary lymph organs where they come in contact with foreign antigens. Germinal centers (GCs) are formed in the lymph follicle, and naïve B cells are displaced from the GCs leading to the formation of an own compartment called the B cell mantle, the differentiation stage at which mantle cell lymphoma occurs. Within the GCs, the B cells actively mutate their BCR to further increase its affinity to the encountered antigen [23, 24, 27].

## The B cell receptor and its involvement in genetic alterations in mature B cell neoplasms

The abovementioned process of genetic recombination is tightly controlled. Nevertheless, erroneous DNA recombination or mutations in checkpoint proteins can result in B cells with the ability to proliferate and eventually form B cell lymphomas [4]. Some of these have specific chromosomal translocations bringing oncogenes under the control of the immunoglobulin (Ig) heavy chain promoter on chromosome 14q32 [4, 8]. In rare cases, these oncogenes juxtapose to the κ-or λ-promoter (on chromosome 2 or 22, respectively) [4, 28]. Since immunoglobulin promoters are highly active in B cells, the translocated oncogenes are overexpressed.

The genetic hallmark in MCL is the chromosomal translocation t(11;14)(q13;q32). This aberration leads to immunoglobulin promoter-driven constitutive expression of the cell cycle regulator Cyclin D1 (encoded by the CCND1 gene), which is usually not expressed in B cells [29]. Cyclin D1 dimerizes with cyclin-dependent-kinases (CDK4/6) which, in turn, phosphorylate the retinoblastoma (Rb) tumor suppressor protein [30]. Phosphorylation inactivates the Rb protein, enabling the cell to switch from the G1- to the S-phase in the cell cycle and to proliferate. Some MCL cases without the specific CCND1 translocation but similar morphological appearance have been described, as well [31–35]. However, these often carry translocations of other cyclin genes like CCND2 or CCND3 [33–35].

Of note, B cells in healthy individuals may also harbor chromosomal translocations like the ones found in B cell lymphoma [36, 37]. The chromosomal aberrations deemed ‘specific’ in the lymphoma B cells are therefore probably only an important first step in lymphoma development, and the interplay of additional mutations are required for the B cell to undergo malignant transformation [33]. In line with this hypothesis, MCL shows a massive dysregulation in the RNA levels of multiple cell cycle-related and anti-apoptotic proteins [33, 38].

As outlined above, the major functional role of a B cell is the expression of a BCR and, upon its terminal differentiation into a plasma cell, the secretion of highly specific immunoglobulins. All B cells keep expressing their clone-specific immunoglobulin throughout the life span of the individual cell. In recent years, it became increasingly clear that the BCR retains its important role for survival and cell proliferation even after transformation of the B cells in many if not most B cell neoplasms [4]. The BCR in lymphoma B cells has received tremendous interest after several studies showed an activated BCR-signaling pathway in these cells, and early clinical studies with BCR pathway inhibitors have yielded very promising results in lymphoma patients (see below). In fact, an increasing body of evidence, gained in recent years, strongly supports the theory that the BCR plays an important functional role in the pathogenesis and progression of several lymphomas. This is particularly well characterized for diffuse large B cell lymphoma (DLBCL) and for chronic lymphocytic leukemia (CLL). For example, the gene expression profiling of lymphoma cells separates DLBCL into two distinct sub-entities—one of them characterized by ongoing BCR signaling [39–41] and consequently designated as activated B cell-like (ABC-) DLBCL [39, 40, 42]. In CLL, the role of the B cell receptor is even more pronounced. CLL cases with mutated immunoglobulins (M-CLL) have a more indolent course of their disease and a more favorable clinical outcome compared to patients with unmutated BCRs (UM-CLL) [43]. Moreover, the immunoglobulin repertoire in CLL B cells is much less diverse than expected if transformation occurred randomly in a given B cell. In the latter scenario, one would expect an almost unlimited diversity of different B cell receptors in CLL with unique BCR rearrangements in all individual patients. However, this is not the case. For example, there are multiple BCR-stereotypes with the same variable heavy chain regions and identical or highly similar CDR3 regions [44]. In addition, CLL can be categorized into only few classes of distinct patterns of epitope recognition [45]. These studies strongly point towards shared epitopes recognized by B cell receptors of different CLL patients [44]. Although not quite as striking as in CLL, studies in MCL have revealed a similar bias with stereotypes in the immunoglobulin repertoire in MCL [46] as described in detail below.

## Mechanisms of BCR-activation in lymphoma

The BCR and BCR signaling configurations in several B cell neoplasms suggest an antigen-driven or otherwise BCR-driven selection of B cell clones during or prior to the process of transformation. Some of the distinct mechanisms of this drive might be specific for certain entities while others could be similar among various entities. In this regard, CLL is the disease investigated in most detail so far. Several distinct epitopes and/or antigens recognized by CLL BCRs have been described. Most of them are autoantigens such as the myosin heavy chain IIA, vimentin and neoantigens generated by oxidation of proteins [45, 47–51]. These findings imply that most, if not all, CLL cells derive from autoreactive B cells. This might link lymphomas to systemic autoimmune disorders [47]. A subset of MCL samples also showed autoantigen binding (see below).

In addition, a large proportion of CLL cells show cell-autonomous BCR signaling induced by self-recognition of the BCR [52, 53]. This unusual cell activation mechanism seems to be a unique feature of CLL cells and has not been described in other entities so far. Other lymphoma subtypes may use different ways of BCR signaling activation instead, such as mutated CD79 ITAMs which result in the formation of BCR clusters similar to activated BCRs and thus also maintain a chronic active BCR signaling, as described in about 20% of ABC-DLBCL [41]. MCL cells, however, show no autonomous signaling and harbor no mutations in the CD79 domains [54].

Follicular lymphoma cells show highly mutated immunoglobulin sequences with an acquisition of N-glycosylation sites in the antigen-binding sites [55–57]. Normally, the introduction of N-glycosylation sites is a potential mechanism for a B cell to recover from self-reactivity [58] but the introduced N-glycans might also be bound by opportunistic bacteria [59]. Although the MCL-derived BCRs show no enrichment of N-glycosylation sites, an infection-associated lymphoma development is a conceivable scenario in all lymphomas including MCL. Even the development of autoimmune diseases and therefore the development of autoreactive CLL cells can be linked to encountered infections [60–62].

## Functional involvement of the B cell receptor in mantle cell lymphoma

Due to the low frequency of MCL with the resulting lack of large cohorts and patient sample repositories, the current knowledge on MCL BCRs is more limited than in CLL, follicular lymphoma (FL), or DLBCL. Thus, the antigens of MCL BCRs or the general mechanisms of their activation are very incompletely understood. Phosphoproteomic analyses revealed that the BCR signaling pathways are active in MCL cells and inhibition of key molecules of these pathways triggers apoptosis in MCL cells in vitro [63]. An ongoing BCR signaling was also found in MCL samples in vivo [64]. Moreover, BCR signaling inhibitors like the Bruton tyrosine kinase (BTK) inhibitor ibrutinib showed very promising efficacy in MCL patients (see below) which further suggest an important and ongoing role of the BCR in MCL [65]. Recently, single cell profiling studies revealed that MCL cells showed an increased phosphorylation of multiple BCR pathway molecules, like AKT and STAT [66, 67]. Triggering BCR activation led to very strong BCR signaling in MCL cells, but not in CLL and healthy B cells, further highlighting the prominent role of the BCR in MCL. Of note, the phosphorylation patterns and the α-BCR-induced signaling in MCL showed a strong interpatient variability and correlate inversely with susceptibility to BTK and spleen tyrosine kinase (SYK) inhibitors in MCL [67].

**Fig. 1:**
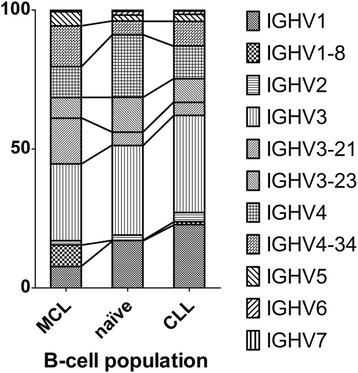
IGHV-gene distribution of MCL-, CLL- and naïve B cell-derived antigen receptors. Studies of the MCL BCRs revealed a biased immunoglobulin repertoire. The differences in the amount of the most abundant IGHV-genes in comparison to CLL and naïve B cells are highlighted with lines between the bars. Data are based on refs [41, 42, 111]

Despite the differences in the phosphorylation pattern, MCL and CLL patients seem to benefit more from BCR signaling inhibitors than other entities like follicular lymphoma [65]. It is therefore reasonable to assume that these diseases might share more similarities, and some of the findings on the BCRs in CLL might also be observed in MCL. In line with this hypothesis, MCL BCRs show similar redundancies and stereotypies as CLL BCRs, even though in a lower proportion of cases [46]. This clearly points towards an antigen-driven lymphomagenesis in both entities. However, the BCR subsets observed in MCL are different from the subsets described in CLL **(**Fig. 1 **)**. In fact, only four Ig heavy chain genes (in order of their abundance: IGHV3–21, IGHV4–34, IGHV1–8, and IGHV3–23) are found in almost half of all MCL-derived BCRs [46]. The isotype distribution of the light chain is biased as well, with a lambda/kappa ratio of about 2:1, representing an inversed ratio to what is found in normal B cell populations (lambda/kappa: 1:2) [68–70]. As a result of the specific expansion of a single cell clone expressing only one distinct BCR, it is also possible to determine the light chain corresponding to the identified heavy chain of the lymphoma-derived immunoglobulin in tissue samples. Although only a few studies focused on MCL light chains, the analysis of heavy and light chain pairings revealed a possible MCL subtype which is characterized by the distinct expression of the IGHV3–21 gene together with the IGLV3–19-gene [71]. MCL patients of this subtype seem to have a slightly better prognosis than patients with different MCL-derived BCRs [71]. The reason for this difference remains unknown so far but, once more, shows the heterogeneity of this disease.

Compared to other B cell lymphomas, the mutational load of MCL-derived immunoglobulins is low. Several studies showed that only a subset of 20–29% of MCL harbor immunoglobulins with more than 2% deviation from the germline sequence [46, 72–74]. In CLL, this 2% cutoff was often used to distinguish between mutated and unmutated CLL, with marked prognostic implications (see above) [43, 75]. In MCL, however, the usefulness of this cutoff remains questionable and does not seem to be applicable. Hadzidimitriou and colleagues proposed a more detailed differentiation. They showed that 29.5% of all MCL-derived Ig heavy chains are completely unmutated (which has not been described to this extent in any other lymphoma) and only 13.8% showed more than 3% deviation from the germline sequence [46, 76]. The difference in the mutational load of the BCR has led to the assumption that MCL develops from two different pathways. The classical MCL derives from SOX11-positive cells with unmutated or minimally mutated IGHVs and shows a more aggressive behavior. The leukemic non-nodal MCL, on the other hand, develops from IGHV-mutated SOX-negative B cells and usually has a more indolent course [7, 38].

There is little knowledge on potential ongoing changes in the BCR once MCL has developed into a clinically detectable disease. Towards this end, we recently analyzed the MCL-derived immunoglobulin repertoire of two sequential biopsies of the same patient by next-generation sequencing (unpublished data). We saw virtually no ongoing mutations in the analyzed MCL-derived Ig sequences over a 4-year period, which is in great contrast to observations made in follicular lymphoma with an ongoing mutation pattern of the FL-derived immunoglobulins over time [77]. However, the molecular pathogenesis of FL and MCL differs profoundly and FL-derived immunoglobulin rearrangements always have a very high mutational load. Although our observation was made only in a single patient, it might indicate that even minor mutations in the MCL-BCR could diminish the B cells’ ability to proliferate and might therefore be negatively selected. Nevertheless, further analysis of a missing or ongoing mutation of MCL immunoglobulins, and lymphoma immunoglobulins in general, is needed to foster our understanding of immunoglobulin stability in these diseases. High-throughput methods like next-generation sequencing will help in the analysis of the changes which occur in the different B cell lymphomas entities over time.

Unmutated (UM) immunoglobulins are often regarded as polyreactive, and it was shown that UM-CLL-derived immunoglobulins bind to autoantigens presented by HEp-2 cells [48]. A similar study with MCL-derived immunoglobulins demonstrated that about one third of all MCL-derived immunoglobulins bind HEp-2 antigens [49], an observation which is confirmed by our group (unpublished data). However, this amount of autoreactive immunoglobulins in MCL is lower than the observed amount in M-CLL (approximately 56.7%) and much lower compared to UM-CLL cells which expressed autoreactive BCRs in 89.6% of all cases [48]. In fact, the observed HEp-2 reactivity of MCL-derived immunoglobulins is comparable to the HEp-2 reactivity of immature B cells (approximately 40%) and is therefore slightly higher than the rate in naïve B cells (approximately 20%) [78]. Nevertheless, we think that these results should be interpreted with caution as they do not necessarily prove the complete absence of autoreactive B cells in two thirds of all MCL patients. Alternatively, MCL BCRs may have a very low affinity, which would result in false-negative experiments, or may bind to autoantigens not expressed in HEp-2 cells. In light of a study which observed varying activity of the activation-induced cytidine deaminase (AID), it seems that the tumor microenvironment plays a crucial role in MCL development which is not sufficiently represented by a single cell line like HEp-2 cells [30, 79]. The influence of the microenvironment during lymphoma development is further highlighted in a study showing a biased usage of the IGHV1–8 gene in splenic MCL cases compared to nodal and extranodal cases [80]. The observed bias in the immunorepertoire might represent a distinct immunopathogenic and antigen selection process in splenic MCLs.

## Superantigenic B cell receptor interaction as a potential pathogenic factor in mantle cell lymphoma

**Fig. 2:**
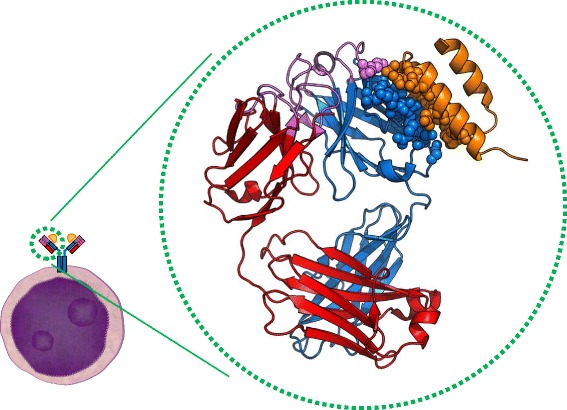
Cartoon representation of an IGHV3-Fab domain with the Domain D of *Staphylococcus aureus* protein A (SpA)**.** Schematic depiction of the BCR on a B cell (left) and the crystallographic structure of its human Fab fragment in association with SpA (in the circle). The heavy chain is shown in blue, the light chain is shown in red and the antigen-binding site with all CDRs is highlighted in purple. In addition, the amino acids which are necessary for the interaction of SpA (orange) with the Fab are depicted by spheres. Note that all but one amino acid are located in the framework region of the Fab. Neither the light chain nor the antigen-binding site contributes to SpA binding. Image adapted from the crystallographic structure published previously [78]. PDB: 1DEE

As an alternative to classical antigens which are bound by the antigen-binding site of the BCR, recent research proposed an involvement of superantigens in MCL development [76]. Superantigens were first described for T cell receptors and represent proteins which bind to the framework regions (FR) of TCRs and BCRs, instead of being bound by the complementarity determining regions (CDRs) [81, 82]. Since the FRs are necessary for the structural integrity of the immunoglobulins, they are far less variable than CDRs. As a result, superantigens can stimulate multiple T or B cells harboring similar variable domains but not necessarily recognize the same epitope or even antigen. Over the years, several superantigens were identified that bind to different amino acid motifs in the variable domains of BCRs (reviewed in [82]). One of the best-characterized immunoglobulin-binding superantigens is the *Staphylococcus aureus* protein A (SpA) [83, 84]. *Staphylococcus aureus* is a common pathogen. Up to 50%, the healthy population is temporarily and about 20% are persistently colonized with this bacterium [85, 86]. Protein A is a well-known protein in molecular biology research labs due to its strong affinity to the constant domain of IgGs and thus its usefulness during the purification of antibodies. Like most superantigens, SpA is probably expressed by *S. aureus* to evade the host immune defense by binding the antibodies at the ‘wrong site’ and therefore thwart the effector function of the immunoglobulin. However, in addition to the well-known ability of SpA to bind the Fc-part of the antibody, it can bind a clearly defined motif in the FR of immunoglobulins (Fig. 2). This binding motif consists of 13 amino acids at specific positions in the variable immunoglobulin domain (represented as spheres in Fig. 2), which is present in nearly all immunoglobulins with the IGHV3-family [83]. SpA binding can crosslink the membrane-bound BCRs without occupying their specific antigen-binding site which can be seen in Fig. 2. Earlier studies have shown that stimulation of human blood cells with SpA in vitro leads to a biased immunoglobulin repertoire and induces selective proliferation of IGHV3-expressing B cells [87]. Importantly, the IGHV3-gene family is the most abundant IGHV-family and about half of all MCL- and CLL-cells express an IGHV3-gene. Nearly every MCL-BCR expressing an IGHV3 immunoglobulin also presents the SpA motif, and it was shown that these BCRs can be activated by SpA [76]. In healthy and matured B cells, the SpA motif is often mutated and the BCR cannot be activated by SpA anymore. Given the low mutational load and the biased usage of certain immunoglobulin genes like the IGHV3–21-gene in MCL, it seems to be a reasonable assumption that superantigens in general and SpA in particular might play an important role in the development and/or progression of MCL. Moreover, the intact SpA binding motif is also present in other entities like Burkitt lymphoma and CLL, raising the question whether different lymphoma entities might be caused by such triggers as well [88, 89]. Although merely hypothetical at this point, a superantigenic activation of a very large amount of early B cells appears to be a plausible first step in the development of lymphomas in general.

On the other hand*,* in vivo experiments showed a strong decrease of B cells expressing the IGHV3-gene after SpA exposition which is probably a result of the increased B cell proliferation and the concomitant overconsumption of cytokines and the lack of secondary signals [90]. However, early lymphoma B cells might overcome this lack of signals as a result of previous mutations, and since whole B cell subpopulations are activated and proliferated, certain already mutated B cells might escape apoptosis and eventually transform into neoplasia. Although highly speculative at this point, the outlined superantigen-dependent lymphoma development could be an additional path in lymphomagenesis, besides the ones described above such as the cell-autonomous signaling in CLL and the—also infection-associated—development of FL via bacterial lectins. Multiple further superantigens are known that are able to bind to immunoglobulins from MCL, CLL, and Burkitt lymphoma [46, 88, 89]. These include the carbohydrate I/i (binding to IGHV4–34) and the *Peptostreptococcus magnus* protein L (binding to κ-light chains) [91, 92].

Despite these advances in understanding, more research is necessary to evaluate if an ongoing infection promotes or is needed for lymphoma progression, if the eradication of the infection may improve clinical outcome, or if a single superantigenic trigger might be sufficient for lymphoma development followed by other B cell activation mechanisms promoting lymphoma progression.

## Targeting the BCR signaling cascade in MCL

The introduction of the anti-CD20 antibody rituximab almost two decades ago has tremendously altered the treatment paradigms of mature B cell lymphoma [93, 94]. This has remained the biggest advancement in lymphoma therapy in a very long time, making it part of the standard treatment in all CD20-positive lymphomas (i.e., the majority of lymphomas). Although rituximab is also effective in MCL and enhances its sensitivity towards chemotherapy [95], MCL continues to have a prognosis considerably worse than most other lymphomas. And this is despite recent advances in upfront treatment (see above) and inclusion of treatment algorithms such as high-dose chemotherapy and stem cell transplantation into first line therapy settings that are used only in the relapsed or refractory situation in other lymphoma entities. Except for the few indolent forms of MCL, the majority of patients relapse within years after initial treatment and treatment options have been limited in this situation. The recognition of the role of the BCR in the pathobiology of several lymphoma entities has also prompted the development of a class of novel drugs with profound activity in these diseases. In consequence, inhibition of the BCR downstream signaling cascade has evolved as a promising new treatment option (reviewed in [65]), but not for all lymphomas alike. Most data exist on ibrutinib which inhibits Bruton tyrosine kinase (see below). The observed activity in clinical trials on various lymphoma entities ranges from efficacy in almost all patients such as in CLL [96] to hardly any significant activity as a monotherapy in GCB (germinal center B cell like) subtypes of DLBCL [97]. In view of the fact that most MCL cells appear to depend on BCR signaling, it has been pertinent to test BCR signaling inhibition in this entity. In fact, it has turned out that this approach is a major step forward in the treatment of relapsed and refractory patients and in the future maybe as part of the first line treatment in MCL (see below).

**Fig. 3:**
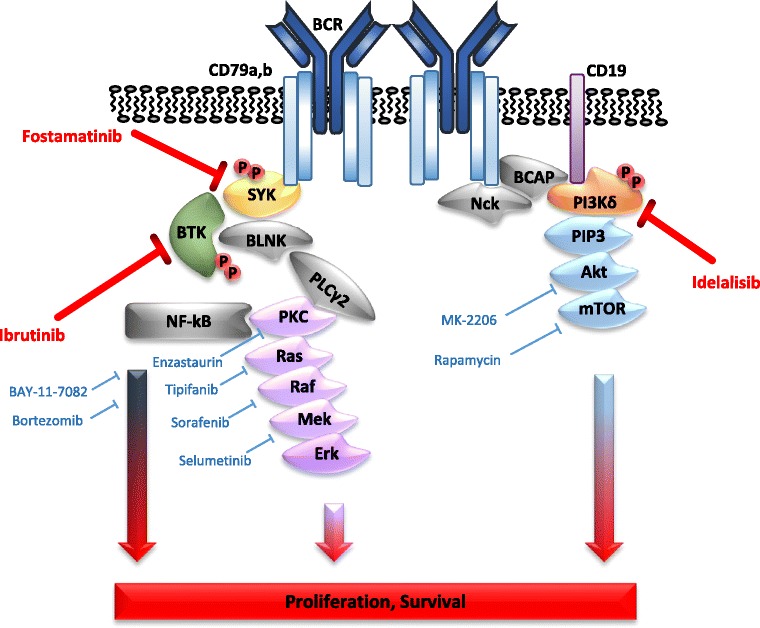
Direct and indirect targeting of the B cell receptor signaling pathway. Direct inhibition of BCR signaling is highlighted in red, potential additional and/or synergistic inhibition strategies with available drugs or drugs in advanced clinical development are shown in blue. Only one example per class of inhibitors is shown

There are several key molecules involved in BCR signaling (Fig. 3). After BCR crosslinking and subsequent phosphorylation of the CD79 ITAMs, the spleen tyrosine kinase (SYK) is recruited to the ITAMs. Thus, this first step in the BCR signaling cascade is the first potential drug target to block B cell proliferation [98]. Interestingly, SYK is overexpressed in many clinical cases of MCL and in several MCL cell line models and SYK inhibition leads to apoptosis induction in vitro, which is particularly strong in cells with high SYK expression [63, 99]. However, in an early phase clinical study, SYK-inhibition did not yield the expected efficacy and resulted in only limited objective response rates (ORR), especially compared to other BCR-inhibitors in CLL patients [100].

Another key element in the BCR-signaling cascade is BTK. This kinase directly affects B cell differentiation and proliferation and thus is a valuable target for inhibition [101]. In addition, BTK is overexpressed in MCL and CLL cells [102, 103]. Ibrutinib is a highly selective BTK inhibitor. It binds covalently to Cys-481 of BTK, leading to an irreversible inhibition of its kinase activity [104]. Several clinical trials showed very promising response rates of ibrutinib in patients with MCL and CLL [105–107], making these two entities paradigmatic for clinical benefits of BCR signaling inhibition. In a pivotal phase II trial, the BTK inhibitor achieved a response rate of 68% (CR 21%) in heavily pretreated MCL patients [105]. Also ibrutinib has been shown to be superior to a previously established MCL salvage treatment, with the mTOR inhibitor temsirolimus. In this trial, it achieved considerably better response rates (72 vs. 40%) and median progression-free survival was markedly improved (14.6 vs 6.2 months) [108]. Consequently, BCR-targeted therapeutic concepts have been adapted as one of the standard regimens in relapsed MCL [12]. Nevertheless, approximately one third of the MCL patients did not respond to ibrutinib treatment. Also, multiple MCL cell lines are intrinsically resistant against this drug in vitro and early progressions under ibrutinib monotherapy with a very aggressive course in the clinical setting have been observed [30, 109, 110]. This resistance might be related to the strong interpatient molecular variability of the BCR activation pattern in MCL characterized previously [67]. Some MCL cells may activate the NF-κB-pathway through the BCR-independent NIK kinase pathway, which in turn might be an additional treatment target [109]. Nevertheless, ibrutinib has been approved as a treatment option after failure of previous therapy in MCL and is currently probably the most widely applied targeted treatment strategy in this setting. Ongoing clinical trials such as the TRIANGLE trial (ClinicalTrials.gov; NCT02858258) also evaluate BTK inhibition as part of the intensive multimodal front line therapy in MCL, and in view of the novel understanding of MCL pathobiology, we believe it to be very likely that the results of such trials will be positive.

Also, since nearly every downstream signaling molecule in the BCR pathway could be the ‘Achilles heel’ of the lymphoma, further targets are being evaluated in preclinical and clinical studies. For instance, inhibitors of PI3K, PKC and AKT are currently under development and tested for their effectiveness [101, 111–113]. In vitro results suggest a significant synergy of combined approaches targeting the BCR pathway.

## Conclusions

**Fig. 4:**
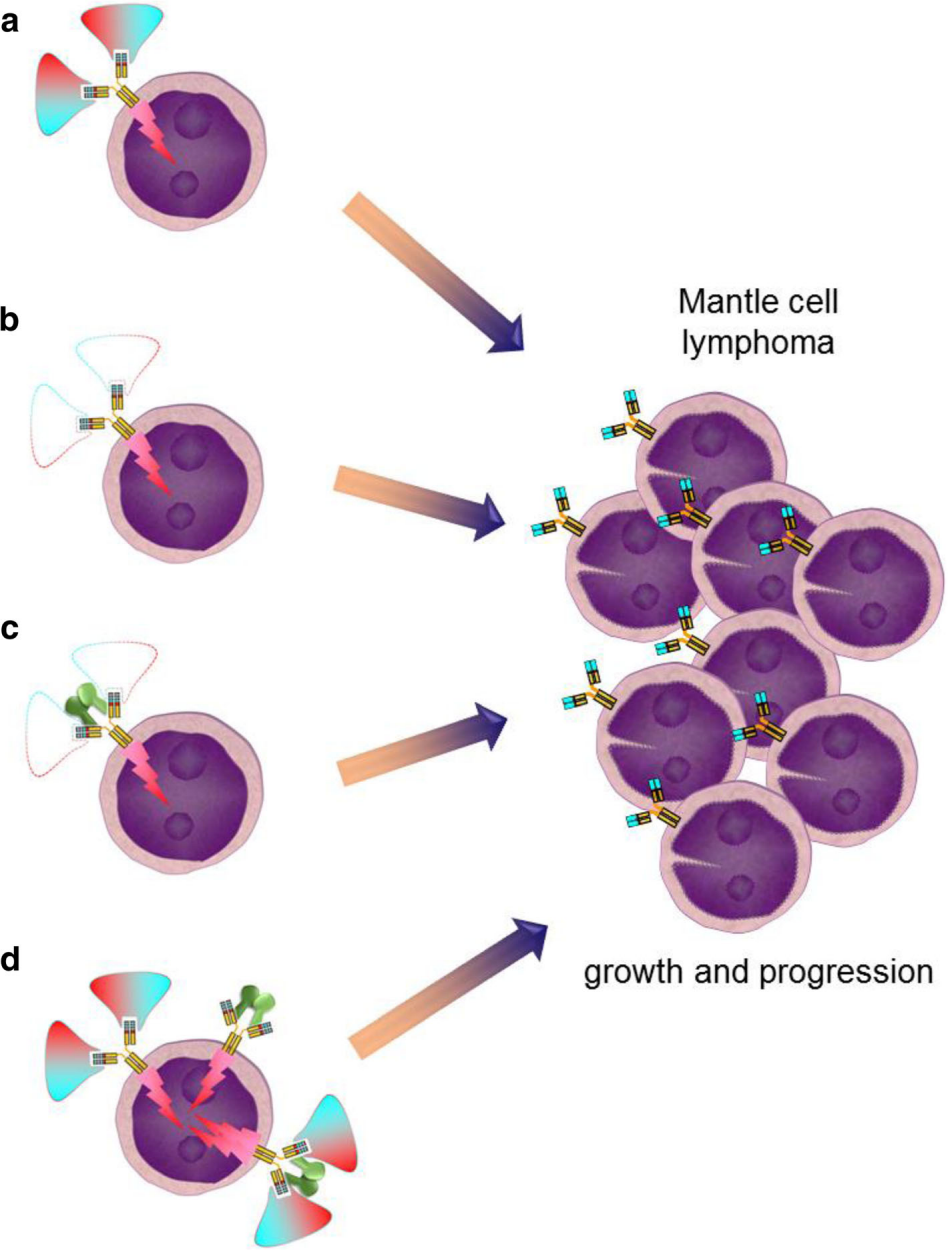
Four potential ways of B cell receptor activation in the pathogenesis of mantle cell lymphoma. a Antigen binding to the CDR3 of the BCR. b Antigen-independent autologous signaling of the BCR. c Superantigen-triggered BCR activation. d Superantigens facilitating BCR activation by classical may be low affinity antigen binding or CD79 aberrations

In the past 20 years, our knowledge about the molecular similarities and differences of the lymphoma entities has greatly increased. As outlined above, this has led to the development of novel treatment options and an improved survival of lymphoma patients. Nevertheless, not all patients seem to benefit from these new agents and the potential to predict outcome after certain treatments is limited. Although established prognostic clinical scores like the Mantle Cell Lymphoma International Prognostic Index [114, 115] or certain molecular features help to guide intensity of front line treatment, there continues to be a need for more personalized therapy of lymphoma in MCL. More research is required to identify the various causes of resistance to the various treatments like ibrutinib in MCL. Besides the urgent need for new predictive biomarkers, it is important to further deepen our understanding of how the different lymphomas develop in the first place. The analysis of the BCR repertoire in mature B cell neoplasms points towards an antigen involvement in the genesis of several lymphomas which might even reveal the opportunity to prevent the actual tumor development. However, despite recent advances such as the discovery of superantigens activating MCL BCRs, there is still too little knowledge about potential BCR-interacting antigens in MCL cells not harboring superantigen-binding sites. While a few MCL BCRs might bind to autoantigens similar to binding patterns described in CLL, the activation mechanisms of other MCL BCRs remains elusive. In principle, there appear to be four potential ways of triggering an activated B cell receptor in the pathogenetic course towards mantle cell lymphoma development: (i) “classical antigenic drive” by antigen binding to the CDRs of the BCR, (ii) antigen-independent autologous signaling of the BCR, e.g., by aberrations within CD79, (iii) superantigen-triggered BCR activation, or (iv) a combination of (i) and (iii) with superantigens facilitating BCR activation by low level (may be due to low affinity) antigen binding or CD79 aberrations (Fig. 4). Future studies have to clarify at which time point in lymphomagenesis the antigenic stimulus takes place and whether it might be compensated by other low affinity interactions during later stages of lymphoma development. Answering these questions will further improve the perspective towards the cure of an increasing percentage of MCL patients in the near future.

## Abbreviations

ABC: Activated B cell like
AID: Activation-induced cytidine deaminase
AKT: Protein encoded by the Act1 gene
BCR: B cell receptor
BTK: Bruton tyrosine kinase
CCND1, 2, 3: Gene encoding cyclin D1, D2, or D3, respectively
CDK: Cyclin-dependent kinase
CDR: Complementarity determining region
CLL: Chronic lymphocytic leukemia
DLBCL: Diffuse large B cell lymphoma
DNA: Deoxyribonucleic acid
FL: Follicular lymphoma
FR: Framework region
GC: Germinal center
GCB: Germinal center B cell like
Ig: Immunoglobulin
IGHV: Immunoglobulin heavy chain variable
ITAMs: Immunoreceptor tyrosine-based activation motifs
MCL: Mantle cell lymphoma
M-CLL: Chronic lymphocytic leukemia with mutated immunoglobulins
mTOR: Mammalian target of rapamycin
NF-κB: Nuclear factor kappa-light-chain-enhancer of activated B cells
NIK: NF-kappa-B-inducing kinase
ORR: Objective response rate
PI3K: Phosphoinositol kinase
PKC: Protein kinase C
Rb: Retinoblastoma
R-CHOP: Rituximab, cyclophosphamide, doxorubicin, vincristine, and prednisone
R-DHAP: Rituximab, dexamethasone, high-dose AraC, cisplatin
R-Hyper-CVAD/MA: Rituximab with cyclophosphamide, doxorubicine, vincristine, dexamethasone, methotrexate, AraC
SpA: *Staphylococcus aureus* protein A
SYK: Spleen tyrosine kinase
TCR: T cell receptor
UM-CLL: Chronic lymphocytic leukemia with unmutated immunoglobulins

## References

[1] Janeway CA, Medzhitov R (2002). Innate immune recognition. Annu Rev Immunol.

[2] Cooper MD, Alder MN (2006). The evolution of adaptive immune systems. Cell.

[3] Tonegawa S (1983). Somatic generation of antibody diversity. Nature.

[4] Küppers R (2005). Mechanisms of B-cell lymphoma pathogenesis. Nat Rev Cancer.

[5] Haematological Malignancy Research Network (HMRN). 2017. https://www.hmrn.org/statistics/incidence. Accessed 12 Oct 2017.

[6] Jemal A, Siegel R, Xu J, Ward E (2010). Cancer statistics, 2010. CA Cancer J Clin.

[7] Swerdlow SH, Campo E, Pileri SA, Harris NL, Stein H, Siebert R (2016). The 2016 revision of the World Health Organization classification of lymphoid neoplasms. Blood.

[8] Nogai H, Dörken B, Lenz G (2011). Pathogenesis of non-Hodgkin’s lymphoma. J Clin Oncol.

[9] The Non-Hodgkin’s Lymphoma Classification Project (1997). A clinical evaluation of the international lymphoma study group classification of non-Hodgkin’s lymphoma. Blood.

[10] Vose JM (2015). Mantle cell lymphoma: 2015 update on diagnosis, risk-stratification, and clinical management: mantle cell lymphoma. Am J Hematol.

[11] Tiemann M, Schrader C, Klapper W, Dreyling MH, Campo E, Norton A (2005). Histopathology, cell proliferation indices and clinical outcome in 304 patients with mantle cell lymphoma (MCL): a clinicopathological study from the European MCL network. Br J Haematol.

[12] Dreyling M, Campo E, Hermine O, Jerkeman M, Le Gouill S, Rule S (2017). Newly diagnosed and relapsed mantle cell lymphoma: ESMO Clinical Practice Guidelines for diagnosis, treatment and follow-up. Ann Oncol.

[13] Geisler CH, Kolstad A, Laurell A, Jerkeman M, Räty R, Andersen NS (2012). Nordic MCL2 trial update: six-year follow-up after intensive immunochemotherapy for untreated mantle cell lymphoma followed by BEAM or BEAC + autologous stem-cell support: still very long survival but late relapses do occur. Br J Haematol.

[14] Hermine O, Hoster E, Walewski J, Bosly A, Stilgenbauer S, Thieblemont C (2016). Addition of high-dose cytarabine to immunochemotherapy before autologous stem-cell transplantation in patients aged 65 years or younger with mantle cell lymphoma (MCL younger): a randomised, open-label, phase 3 trial of the European mantle cell lymphoma network. Lancet Lond. Engl..

[15] Romaguera JE, Khouri IF, Kantarjian HM, Hagemeister FB, Rodriguez MA, McLaughlin P (2000). Untreated aggressive mantle cell lymphoma: results with intensive chemotherapy without stem cell transplant in elderly patients. Leuk. Lymphoma..

[16] Dreyling M, Lenz G, Hoster E, Van Hoof A, Gisselbrecht C, Schmits R (2005). Early consolidation by myeloablative radiochemotherapy followed by autologous stem cell transplantation in first remission significantly prolongs progression-free survival in mantle-cell lymphoma: results of a prospective randomized trial of the European MCL network. Blood.

[17] Le Gouill S, Thieblemont C, Oberic L, Moreau A, Bouabdallah K, Dartigeas C (2017). Rituximab after autologous stem-cell transplantation in mantle-cell lymphoma. N Engl J Med.

[18] Lipsky A, Martin P (2017). Bendamustine-rituximab in mantle cell lymphoma. Lancet Haematol.

[19] Tam CS, Khouri IF (2009). Autologous and allogeneic stem cell transplantation: rising therapeutic promise for mantle cell lymphoma. Leuk. Lymphoma..

[20] Reth M (1992). Antigen receptors on B lymphocytes. Annu Rev Immunol.

[21] Hombach J, Tsubata T, Leclercq L, Stappert H, Reth M (1990). Molecular components of the B-cell antigen receptor complex of the IgM class. Nature.

[22] Flaswinkel H, Reth M (1994). Dual role of the tyrosine activation motif of the Ig-alpha protein during signal transduction via the B cell antigen receptor. EMBO J.

[23] Schroeder HW, Cavacini L (2010). Structure and function of Immunoglobulins. J Allergy Clin Immunol.

[24] Honjo T (1983). Immunoglobulin genes. Annu Rev Immunol.

[25] Tinguely A, Chemin G, Péron S, Sirac C, Reynaud S, Cogné M (2012). Cross talk between immunoglobulin heavy-chain transcription and RNA surveillance during B cell development. Mol Cell Biol.

[26] Rajewsky K (1996). Clonal selection and learning in the antibody system. Nature.

[27] Saribasak H, Gearhart PJ (2012). Does DNA repair occur during somatic hypermutation?. Semin Immunol.

[28] Ondrejka SL, Hsi ED (2015). Pathology of B-cell lymphomas: diagnosis and biomarker discovery. Cancer Treat Res.

[29] Uchimaru K, Taniguchi T, Yoshikawa M, Asano S, Arnold A, Fujita T (1997). Detection of Cyclin D1 (bcl-1, PRAD1) overexpression by a simple competitive reverse transcription-polymerase chain reaction assay in t(11; 14)(q13; q32)-bearing B-cell malignancies and/or mantle cell lymphoma. Blood.

[30] Saba N, Wiestner A (2014). Do mantle cell lymphomas have an “Achilles heel”?. Curr Opin Hematol.

[31] Salaverria I, Zettl A, Beà S, Moreno V, Valls J, Hartmann E (2007). Specific secondary genetic alterations in mantle cell lymphoma provide prognostic information independent of the gene expression-based proliferation signature. J Clin Oncol Off J Am Soc Clin Oncol.

[32] Seto M (2013). Cyclin D1-negative mantle cell lymphoma. Blood.

[33] Fu K, Weisenburger DD, Greiner TC, Dave S, Wright G, Rosenwald A (2005). Cyclin D1-negative mantle cell lymphoma: a clinicopathologic study based on gene expression profiling. Blood.

[34] Salaverria I, Royo C, Carvajal-Cuenca A, Clot G, Navarro A, Valera A (2013). CCND2 rearrangements are the most frequent genetic events in cyclin D1(−) mantle cell lymphoma. Blood.

[35] Herens C, Lambert F, Quintanilla-Martinez L, Bisig B, Deusings C, de Leval L (2008). Cyclin D1-negative mantle cell lymphoma with cryptic t(12;14)(p13;q32) and cyclin D2 overexpression. Blood.

[36] Dölken G, Illerhaus G, Hirt C, Mertelsmann R. BCL-2/JH rearrangements in circulating B cells of healthy blood donors and patients with nonmalignant diseases. . J. Clin. Oncol. Off. J. Am. Soc. Clin Oncologia 1996;14:1333–1344. 10.1200/JCO.1996.14.4.1333 8648392

[37] Limpens J, Stad R, Vos C, de Vlaam C, de Jong D, van Ommen GJ (1995). Lymphoma-associated translocation t(14;18) in blood B cells of normal individuals. Blood.

[38] Jares P, Colomer D, Campo E (2012). Molecular pathogenesis of mantle cell lymphoma. J Clin Invest.

[39] Alizadeh AA, Eisen MB, Davis RE, Ma C, Lossos IS, Rosenwald A (2000). Distinct types of diffuse large B-cell lymphoma identified by gene expression profiling. Nature.

[40] Davis RE, Brown KD, Siebenlist U, Staudt LM (2001). Constitutive nuclear factor κB activity is required for survival of activated B cell-like diffuse large B cell lymphoma cells. J Exp Med.

[41] Davis RE, Ngo VN, Lenz G, Tolar P, Young R, Romesser PB (2010). Chronic active B cell receptor signaling in diffuse large B cell lymphoma. Nature.

[42] Lenz G, Staudt LM (2010). Aggressive lymphomas. N Engl J Med.

[43] Hamblin TJ, Davis Z, Gardiner A, Oscier DG, Stevenson FK (1999). Unmutated Ig VH genes are associated with a more aggressive form of chronic lymphocytic leukemia. Blood.

[44] Agathangelidis A, Darzentas N, Hadzidimitriou A, Brochet X, Murray F, Yan X-J (2012). Stereotyped B-cell receptors in one-third of chronic lymphocytic leukemia: a molecular classification with implications for targeted therapies. Blood.

[45] Binder M, Léchenne B, Ummanni R, Scharf C, Balabanov S, Trusch M (2010). Stereotypical chronic lymphocytic leukemia B-cell receptors recognize survival promoting antigens on stromal cells. PLoS One.

[46] Hadzidimitriou A, Agathangelidis A, Darzentas N, Murray F, Delfau-Larue M-H, Pedersen LB (2011). Is there a role for antigen selection in mantle cell lymphoma? Immunogenetic support from a series of 807 cases. Blood.

[47] Catera R, Silverman GJ, Hatzi K, Seiler T, Didier S, Zhang L (2008). Chronic lymphocytic leukemia cells recognize conserved epitopes associated with apoptosis and oxidation. Mol Med Camb Mass.

[48] Hervé M, Xu K, Ng Y-S, Wardemann H, Albesiano E, Messmer BT (2005). Unmutated and mutated chronic lymphocytic leukemias derive from self-reactive B cell precursors despite expressing different antibody reactivity. J Clin Invest.

[49] Cha S-C, Qin H, Kannan S, Rawal S, Watkins LS, Baio FE (2013). Nonstereotyped lymphoma B cell receptors recognize vimentin as a shared autoantigen. J Immunol Baltim Md 1950.

[50] Chu CC, Catera R, Zhang L, Didier S, Agagnina BM, Damle RN (2010). Many chronic lymphocytic leukemia antibodies recognize apoptotic cells with exposed nonmuscle myosin heavy chain IIA: implications for patient outcome and cell of origin. Blood.

[51] Myhrinder AL, Hellqvist E, Sidorova E, Söderberg A, Baxendale H, Dahle C (2008). A new perspective: molecular motifs on oxidized LDL, apoptotic cells, and bacteria are targets for chronic lymphocytic leukemia antibodies. Blood.

[52] Minden MD, Übelhart R, Schneider D, Wossning T, Bach MP, Buchner M (2012). Chronic lymphocytic leukaemia is driven by antigen-independent cell-autonomous signalling. Nature.

[53] Binder M, Müller F, Frick M, Wehr C, Simon F, Leistler B (2013). CLL B-cell receptors can recognize themselves: alternative epitopes and structural clues for autostimulatory mechanisms in CLL. Blood.

[54] Zhang J, Jima D, Moffitt AB, Liu Q, Czader M, Hsi ED (2014). The genomic landscape of mantle cell lymphoma is related to the epigenetically determined chromatin state of normal B cells. Blood.

[55] Zhu D, McCarthy H, Ottensmeier CH, Johnson P, Hamblin TJ, Stevenson FK (2002). Acquisition of potential N-glycosylation sites in the immunoglobulin variable region by somatic mutation is a distinctive feature of follicular lymphoma. Blood.

[56] Radcliffe CM, Arnold JN, Suter DM, Wormald MR, Harvey DJ, Royle L (2007). Human follicular lymphoma cells contain oligomannose glycans in the antigen-binding site of the B-cell receptor. J Biol Chem.

[57] Coelho V, Krysov S, Ghaemmaghami AM, Emara M, Potter KN, Johnson P (2010). Glycosylation of surface Ig creates a functional bridge between human follicular lymphoma and microenvironmental lectins. Proc Natl Acad Sci U S A.

[58] Sabouri Z, Schofield P, Horikawa K, Spierings E, Kipling D, Randall KL (2014). Redemption of autoantibodies on anergic B cells by variable-region glycosylation and mutation away from self-reactivity. Proc Natl Acad Sci U S A.

[59] Schneider D, Dühren-von Minden M, Alkhatib A, Setz C, van Bergen CAM, Benkißer-Petersen M (2015). Lectins from opportunistic bacteria interact with acquired variable-region glycans of surface immunoglobulin in follicular lymphoma. Blood.

[60] Anderson LA, Landgren O, Engels EA (2009). Common community acquired infections and subsequent risk of chronic lymphocytic leukaemia. Br J Haematol.

[61] Landgren O, Gridley G, Check D, Caporaso NE, Morris BL (2007). Acquired immune-related and inflammatory conditions and subsequent chronic lymphocytic leukaemia. Br J Haematol.

[62] Landgren O, Rapkin JS, Caporaso NE, Mellemkjaer L, Gridley G, Goldin LR (2007). Respiratory tract infections and subsequent risk of chronic lymphocytic leukemia. Blood.

[63] Pighi C, Gu T-L, Dalai I, Barbi S, Parolini C, Bertolaso A (2011). Phospho-proteomic analysis of mantle cell lymphoma cells suggests a pro-survival role of B-cell receptor signaling. Cell Oncol Dordr.

[64] Saba NS, Liu D, Herman SEM, Underbayev C, Tian X, Behrend D (2016). Pathogenic role of B-cell receptor signaling and canonical NF-κB activation in mantle cell lymphoma. Blood.

[65] Jerkeman M, Hallek M, Dreyling M, Thieblemont C, Kimby E, Staudt L, et al. J Intern Med. 2017; 10.1111/joim.12600 28295729

[66] Rudelius M, Pittaluga S, Nishizuka S, Pham TH-T, Fend F, Jaffe ES (2006). Constitutive activation of Akt contributes to the pathogenesis and survival of mantle cell lymphoma. Blood.

[67] Myklebust JH, Brody J, Kohrt HE, Kolstad A, Czerwinski DK, Wälchli S (2017). Distinct patterns of B-cell receptor signaling in non-Hodgkin lymphomas identified by single-cell profiling. Blood.

[68] Bertoni F, Ponzoni M (2007). The cellular origin of mantle cell lymphoma. Int J Biochem Cell Biol.

[69] Bertoni F, Zucca E, Genini D, Cazzaniga G, Roggero E, Ghielmini M (1999). Immunoglobulin light chain kappa deletion rearrangement as a marker of clonality in mantle cell lymphoma. Leuk Lymphoma.

[70] Schraders M, Oeschger S, Kluin PM, Hebeda K, Schuuring E, Groenen PJTA (2009). Hypermutation in mantle cell lymphoma does not indicate a clinical or biological subentity. Mod Pathol.

[71] Walsh SH, Thorsélius M, Johnson A, Söderberg O, Jerkeman M, Björck E (2003). Mutated VH genes and preferential VH3-21 use define new subsets of mantle cell lymphoma. Blood.

[72] Navarro A, Clot G, Royo C, Jares P, Hadzidimitriou A, Agathangelidis A (2012). Molecular subsets of mantle cell lymphoma defined by the IGHV mutational status and SOX11 expression have distinct biologic and clinical features. Cancer Res.

[73] Thorsélius M, Walsh S, Eriksson I, Thunberg U, Johnson A, Backlin C (2002). Somatic hypermutation and VH gene usage in mantle cell lymphoma. Eur J Haematol.

[74] Camacho FI, Algara P, Rodríguez A, Ruíz-Ballesteros E, Mollejo M, Martínez N (2003). Molecular heterogeneity in MCL defined by the use of specificV H genes and the frequency of somatic mutations. Blood.

[75] Damle RN, Wasil T, Fais F, Ghiotto F, Valetto A, Allen SL (1999). Ig V gene mutation status and CD38 expression as novel prognostic indicators in chronic lymphocytic leukemia. Blood.

[76] Fichtner M, Spies E, Seismann H, Riecken K, Engels N, Gosch B (2016). Complementarity determining region-independent recognition of a superantigen by B-cell antigen receptors of mantle cell lymphoma. Haematologica.

[77] Loeffler M, Kreuz M, Haake A, Hasenclever D, Trautmann H, Arnold C (2015). Genomic and epigenomic co-evolution in follicular lymphomas. Leukemia.

[78] Wardemann H, Yurasov S, Schaefer A, Young JW, Meffre E, Nussenzweig MC (2003). Predominant autoantibody production by early human B cell precursors. Science.

[79] Xochelli A, Sutton L-A, Agathangelidis A, Stalika E, Karypidou M, Marantidou F (2015). Molecular evidence for antigen drive in the natural history of mantle cell lymphoma. Am J Pathol.

[80] Pouliou E, Xochelli A, Kanellis G, Stalika E, Sutton L-A, Navarro A (2017). Numerous ontogenetic roads to mantle cell lymphoma: Immunogenetic and Immunohistochemical evidence. Am J Pathol.

[81] Huber BT, Hsu PN, Sutkowski N (1996). Virus-encoded superantigens. Microbiol Rev.

[82] Silverman GJ, Goodyear CS (2006). Confounding B-cell defences: lessons from a staphylococcal superantigen. Nat. Rev. Immunol..

[83] Graille M, Stura EA, Corper AL, Sutton BJ, Taussig MJ, Charbonnier JB (2000). Crystal structure of a Staphylococcus aureus protein a domain complexed with the Fab fragment of a human IgM antibody: structural basis for recognition of B-cell receptors and superantigen activity. Proc Natl Acad Sci U S A.

[84] Tashiro M, Montelione GT (1995). Structures of bacterial immunoglobulin-binding domains and their complexes with immunoglobulins. Curr Opin Struct Biol.

[85] Lowy FD (1998). Staphylococcus aureus infections. N Engl J Med.

[86] van Belkum A, Melles DC, Nouwen J, van Leeuwen WB, van Wamel W, Vos MC (2009). Co-evolutionary aspects of human colonisation and infection by Staphylococcus aureus. Infect Genet Evol.

[87] Kristiansen SV, Pascual V, Lipsky PE (1994). Staphylococcal protein A induces biased production of Ig by VH3-expressing B lymphocytes. J. Immunol. Baltim. Md 1950.

[88] Baptista MJ, Calpe E, Fernandez E, Colomo L, Cardesa-Salzmann TM, Abrisqueta P (2014). Analysis of the IGHV region in Burkitt’s lymphomas supports a germinal center origin and a role for superantigens in lymphomagenesis. Leuk Res.

[89] Murray F, Darzentas N, Hadzidimitriou A, Tobin G, Boudjogra M, Scielzo C (2008). Stereotyped patterns of somatic hypermutation in subsets of patients with chronic lymphocytic leukemia: implications for the role of antigen selection in leukemogenesis. Blood.

[90] Goodyear CS, Silverman GJ (2003). Death by a B cell superantigen: in vivo VH-targeted apoptotic supraclonal B cell deletion by a staphylococcal toxin. J Exp Med.

[91] Graille M, Stura EA, Housden NG, Beckingham JA, Bottomley SP, Beale D (2001). Complex between Peptostreptococcus magnus protein L and a human antibody reveals structural convergence in the interaction modes of Fab binding proteins. Struct Lond Engl 1993.

[92] Potter KN, Hobby P, Klijn S, Stevenson FK, Sutton BJ (2002). J. Immunol. Baltim. Md 1950.

[93] Maloney DG, Grillo-López AJ, White CA, Bodkin D, Schilder RJ, Neidhart JA (1997). IDEC-C2B8 (Rituximab) anti-CD20 monoclonal antibody therapy in patients with relapsed low-grade non-Hodgkin9s lymphoma. Blood.

[94] Dotan E, Aggarwal C, Smith MR (2010). Impact of Rituximab (Rituxan) on the treatment of B-cell non-Hodgkin’s lymphoma. Pharm Ther.

[95] Lenz G, Dreyling M, Hoster E, Wörmann B, Dührsen U, Metzner B (2005). Immunochemotherapy with rituximab and cyclophosphamide, doxorubicin, vincristine, and prednisone significantly improves response and time to treatment failure, but not long-term outcome in patients with previously untreated mantle cell lymphoma: results of a prospective randomized trial of the German low grade lymphoma study group (GLSG). J Clin Oncol Off J Am Soc Clin Oncol.

[96] Burger JA, Tedeschi A, Barr PM, Robak T, Owen C, Ghia P (2015). Ibrutinib as initial therapy for patients with chronic lymphocytic leukemia. N Engl J Med.

[97] Wilson WH, Young RM, Schmitz R, Yang Y, Pittaluga S, Wright G (2015). Targeting B cell receptor signaling with ibrutinib in diffuse large B cell lymphoma. Nat Med.

[98] Mócsai A, Ruland J, Tybulewicz VLJ (2010). The SYK tyrosine kinase: a crucial player in diverse biological functions. Nat Rev Immunol.

[99] Rinaldi A, Kwee I, Taborelli M, Largo C, Uccella S, Martin V (2006). Genomic and expression profiling identifies the B-cell associated tyrosine kinase Syk as a possible therapeutic target in mantle cell lymphoma. Br J Haematol.

[100] Friedberg JW, Sharman J, Sweetenham J, Johnston PB, Vose JM, LaCasce A (2010). Inhibition of Syk with fostamatinib disodium has significant clinical activity in non-Hodgkin lymphoma and chronic lymphocytic leukemia. Blood.

[101] Buchner M, Müschen M (2014). Targeting the B-cell receptor signaling pathway in B lymphoid malignancies. Curr Opin Hematol.

[102] Cinar M, Hamedani F, Mo Z, Cinar B, Amin HM, Alkan S (2013). Bruton tyrosine kinase is commonly overexpressed in mantle cell lymphoma and its attenuation by Ibrutinib induces apoptosis. Leuk Res.

[103] Herman SEM, Gordon AL, Hertlein E, Ramanunni A, Zhang X, Jaglowski S (2011). Bruton tyrosine kinase represents a promising therapeutic target for treatment of chronic lymphocytic leukemia and is effectively targeted by PCI-32765. Blood.

[104] Pan Z, Scheerens H, Li S-J, Schultz BE, Sprengeler PA, Burrill LC (2007). Discovery of selective irreversible inhibitors for Bruton’s tyrosine kinase. ChemMedChem.

[105] Wang ML, Rule S, Martin P, Goy A, Auer R, Kahl BS (2013). Targeting BTK with ibrutinib in relapsed or refractory mantle-cell lymphoma. N Engl J Med.

[106] Hendriks RW, Yuvaraj S, Kil LP (2014). Targeting Bruton’s tyrosine kinase in B cell malignancies. Nat Rev Cancer.

[107] Advani RH, Buggy JJ, Sharman JP, Smith SM, Boyd TE, Grant B, et al. Bruton tyrosine kinase inhibitor ibrutinib (PCI-32765) has significant activity in patients with relapsed/refractory B-cell malignancies. J. Clin. Oncol. Off. J. Am. Soc. Clin Oncologia 2013;31:88–94. 10.1200/JCO.2012.42.7906 PMC5505166 23045577

[108] Dreyling M, Jurczak W, Jerkeman M, Silva RS, Rusconi C, Trneny M (2016). Ibrutinib versus temsirolimus in patients with relapsed or refractory mantle-cell lymphoma: an international, randomised, open-label, phase 3 study. Lancet Lond Engl.

[109] Rahal R, Frick M, Romero R, Korn JM, Kridel R, Chun Chan F (2014). Pharmacological and genomic profiling identifies NF-κB-targeted treatment strategies for mantle cell lymphoma. Nat Med.

[110] Martin P, Maddocks K, Leonard JP, Ruan J, Goy A, Wagner-Johnston N (2016). Postibrutinib outcomes in patients with mantle cell lymphoma. Blood.

[111] Bojarczuk K, Bobrowicz M, Dwojak M, Miazek N, Zapala P, Bunes A (2015). B-cell receptor signaling in the pathogenesis of lymphoid malignancies. Blood Cells Mol Dis.

[112] Kahl BS, Spurgeon SE, Furman RR, Flinn IW, Coutre SE, Brown JR (2014). A phase 1 study of the PI3Kδ inhibitor idelalisib in patients with relapsed/refractory mantle cell lymphoma (MCL). Blood.

[113] Dreyling M, Morschhauser F, Bouabdallah K, Bron D, Cunningham D, Assouline SE (2017). Phase II study of copanlisib, a PI3K inhibitor, in relapsed or refractory, indolent or aggressive lymphoma. Ann Oncol Off J Eur Soc Med Oncol.

[114] Hoster E, Dreyling M, Klapper W, Gisselbrecht C, van Hoof A, Kluin-Nelemans HC (2008). A new prognostic index (MIPI) for patients with advanced-stage mantle cell lymphoma. Blood.

[115] Hoster E, Rosenwald A, Berger F, Bernd H-W, Hartmann S, Loddenkemper C (2016). Prognostic value of Ki-67 index, cytology, and growth pattern in mantle-cell lymphoma: results from randomized trials of the European mantle cell lymphoma network. J Clin Oncol Off J Am Soc Clin Oncol.

